# Prosthetic home intervention induces cortical plasticity in paediatrics with congenital limb reduction

**DOI:** 10.1093/braincomms/fcae044

**Published:** 2024-07-08

**Authors:** Jordan A Borrell, Arun Karumattu Manattu, Christopher Copeland, Kaitlin Fraser, Andrew D’Ovidio, Zach Granatowicz, Liliana Delgado, Jorge M Zuniga

**Affiliations:** Department of Biomechanics, University of Nebraska at Omaha, Omaha, NE 68182, USA; Center for Biomedical Rehabilitation and Manufacturing, University of Nebraska at Omaha, Omaha, NE 68182, USA; Department of Occupational Therapy Education, University of Kansas Medical Center, Kansas City, KS 66103, USA; Department of Biomechanics, University of Nebraska at Omaha, Omaha, NE 68182, USA; Department of Biomechanics, University of Nebraska at Omaha, Omaha, NE 68182, USA; Department of Biomechanics, University of Nebraska at Omaha, Omaha, NE 68182, USA; Department of Biomechanics, University of Nebraska at Omaha, Omaha, NE 68182, USA; Department of Biomechanics, University of Nebraska at Omaha, Omaha, NE 68182, USA; Department of Biomechanics, University of Nebraska at Omaha, Omaha, NE 68182, USA; Department of Biomechanics, University of Nebraska at Omaha, Omaha, NE 68182, USA; Center for Biomedical Rehabilitation and Manufacturing, University of Nebraska at Omaha, Omaha, NE 68182, USA

**Keywords:** fNIRS, congenital limb reduction, hemodynamic response, brain activity, prosthesis training

## Abstract

Paediatrics with congenital upper-limb reduction deficiency often face difficulties with normal development such as motor skills, needing assistance with daily activities such as self-care limitations with certain movements, sports, or activities. The purpose of this non-randomized longitudinal controlled trial was to assess, using intent-to-treat analysis, the effects of an 8-week home intervention of prosthetic use on the sensorimotor cortex in paediatrics with congenital upper-limb reduction deficiency. A paediatric population with congenital upper-limb reduction deficiency (*n* = 14) who were aged 6–18 years and who had a 20° or greater range of motion in the appropriate joint of the affected arm to move the body-powered prosthesis were enrolled. An age- and sex-matched control group (*n* = 14) was also enrolled. Participants were non-randomized and fitted with a custom low-cost 3D printed prosthesis and participated in 8 weeks of prosthetic use training at home. Control participants utilized a prosthetic simulator. The home intervention incorporated daily use training and exercises utilizing the prosthesis in direct use and assistive tasks explained by the researchers. After the home intervention, both groups displayed significant improvements in gross manual dexterity. During prosthetic use with the affected limb, significant increases in oxygenated hemodynamic responses were only displayed in the left premotor cortex of the upper-limb reduction deficiency group. The novel findings of this non-randomized longitudinal controlled trial suggest that the intervention may have improved the functional role of the left hemisphere which translated to the improvement of learning direction during adaptation to visuomotor control. The prosthetic home intervention was assumed to provide closed-loop training which could provide a direct benefit to the motor development of paediatrics with upper-limb reduction deficiency.

## Teaser

Borrell *et al*. report that an 8-week prosthesis home intervention provided benefit to paediatrics with upper-limb reduction deficiency. The behavioural improvements and cortical plasticity suggest a functional role of the left hemisphere to improve performance and the potential embodiment of a prosthetic device in the cerebral cortex after prosthesis training.

## Graphical Abstract

**Graphical Abstract:**
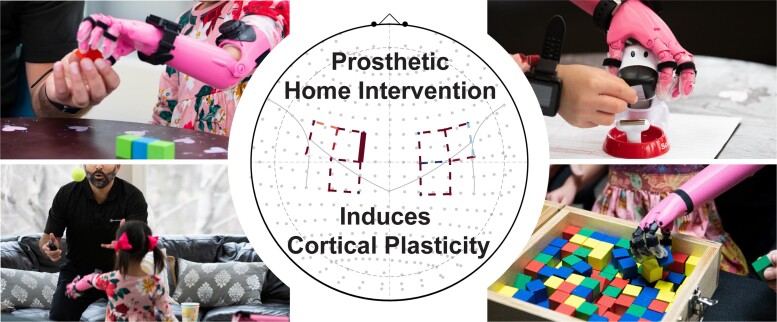

## Introduction

The Centers for Disease Control and Prevention (CDC) reports that approximately 4 out of every 10 000 babies are born with upper-limb reductions every year in the United States.^1,2^ Other parts of the world report this number from 3.4 to 5.3 of 10 000 children.^3^ However, many cases are unreported due to the lack of a mandatory reporting system of birth defects and amputations at a young age. In order to restore function in paediatrics with upper-limb reduction deficiencies (ULRD), the main treatment is the use of a prosthetic device.^1^ However, the main barrier to treatment is the cost of providing a functional prosthesis, which ranges from $4000 to $10 000 for a body-powered prosthesis and $25 000 to $75 000 for an electronically driven prosthesis.^4,5^ Additional barriers to treatment are the continued increased cost and lack of insurance coverage for prosthetic devices, which limit access.^5-7^

The CDC indicated that paediatrics with ULRD will face potential problems including, difficulties with normal development such as motor skills, needing assistance with daily activities such as self-care limitations with certain movements, sports or activities, as well as potential emotional and social issues because of the physical appearance.^1^ Even with efforts to provide 3D printed prostheses for children at a low cost that are lightweight, visually appealing and comfortable,^6-8^ the abandonment and rejection of prosthetic devices, with up to 58% rejection, still remains high.^9-11^ While the factors for rejection are exclusive to the design of the prosthesis, previous literature suggest that the involvement of the specific neuronal control mechanisms that limit the functional of these devices.^9,10,12^ Thus, there is a significant knowledge gap about the neural mechanism underlying the high rejection rate of upper limb prostheses in a paediatric population.^9^ Our previous studies have focused on this knowledge gap to identify the muscle coactivation,^13^ functional changes,^14^ and brain lateralization^15^ in paediatrics with ULRD as they utilize 3D printed, body-powered prostheses.

Further evidence has now established that the two cerebral hemispheres show a considerable degree of motor lateralization, in which the left and right hemispheres are specialized for various aspects of motor control.^16^ This model of motor lateralization indicates that the left hemisphere predicts and accounts for limb dynamics while the right hemisphere stabilizes limb position through impedance control mechanisms, which have been shown in patients with unilateral brain damage.^17-20^ We have additionally conducted preliminary data in a case study which indicated that drastic changes in hemodynamic responses occurred after prosthesis use training.^21^ Thus, the purpose of this study was to expand this case study to a larger, longitudinal clinical trial to increase the impact of these results. We hypothesize that 8 weeks of the prosthesis use home intervention will produce robust changes to the sensorimotor cortex of paediatrics with ULRD. Our secondary hypothesis is that there will be a non-significant difference in gross manual dexterity between device use (i.e. prosthetic and prosthetic simulator) and intact hand use (i.e. non-affected and dominant hand) before the onset of the home intervention.

## Materials and methods

**Figure 1:**
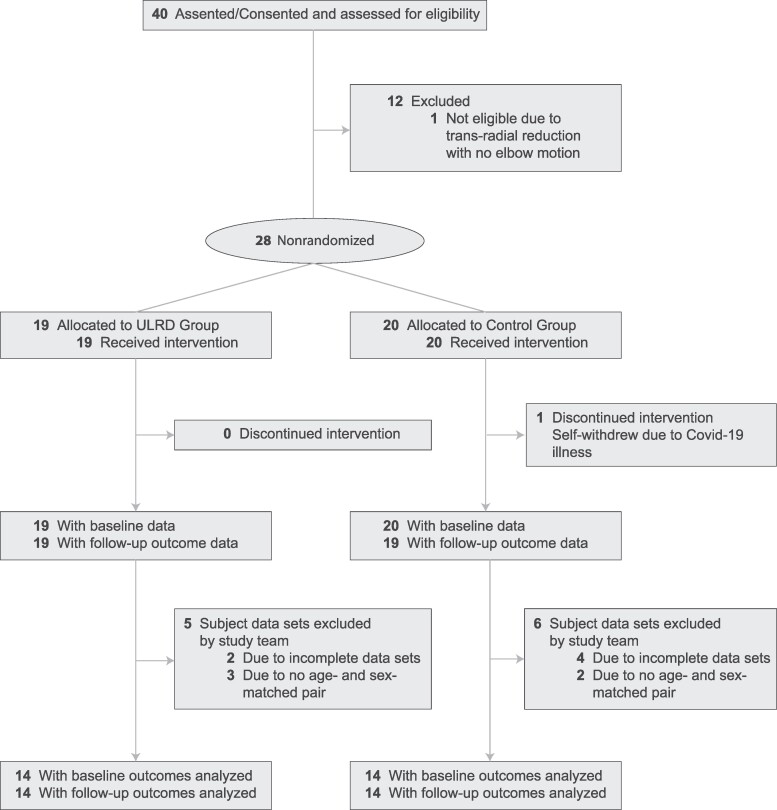
TREND flow diagram.

All parents and their children were informed about the study and parents signed a parental consent. An assent was explained to the paediatric and signed by the children and their parents. In the state of Nebraska, the age of consent to sign a document is 19 years of age, so a parent’s signature was needed for the entire paediatric population in this study. Thus, the paediatric population will hereby be referred to as ‘participants.’ Consent and assent were obtained according to the Declaration of Helsinki. Detailed safety guidelines were given to the parents regarding the use and care of the prosthesis. The study was approved by the University of Nebraska at Omaha Institutional Review Board. The trial was prospectively registered at ClinicalTrials.gov (NCT04110730). This trial followed the Transparent Reporting of Evaluations with Nonrandomized Designs (TREND) reporting guidelines (Fig. 1).

### Outcomes

**Figure 2:**
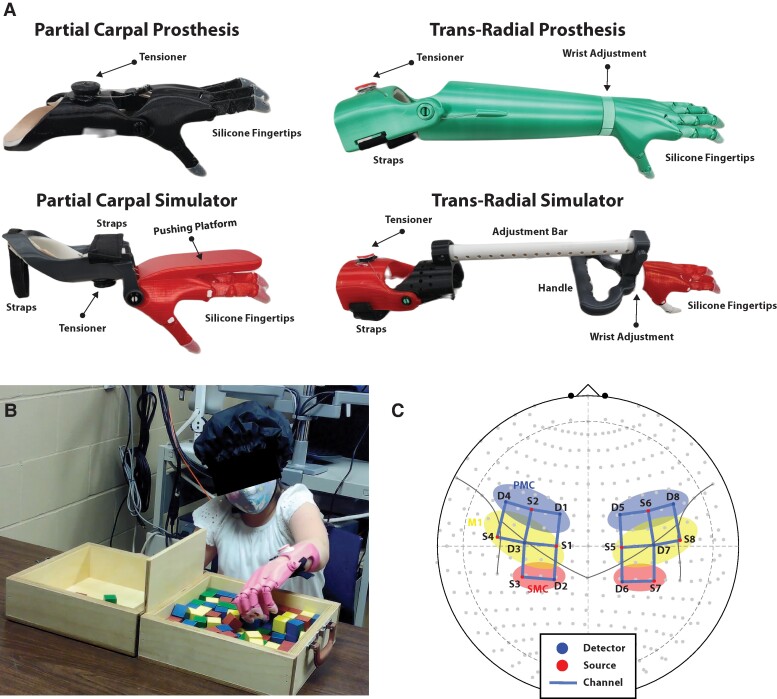
**Experimental design.** (**A**) Description of prostheses and prosthetic simulators. The partial carpal and trans-radial prostheses were utilized by the ULRD group, while the partial carpal and trans-radial simulators were utilized by the Control group. The prosthetic simulators were designed to mimic the control mechanism of the prostheses. The partial carpal simulator allowed the Control group to rest their existing hand on top of the simulator hand, with the wrist in slight extension. A pushing platform placed above the hand allowed wrist active flexion and passive extension to facilitate actuation of the hand. Similarly, the trans-radial simulator incorporates a handle for the Control group to grab and actuate the device by elbow flexion. (**B**) Participant setup during the Box and Blocks Test. (**C**) fNIRS head probe with sources and detectors positioned across the sensorimotor cortices. The regions of interest over the sensorimotor cortex are defined as follows: top channels (blue area) = pre-motor cortex (PMC); middle channels (yellow area) = primary motor cortex (M1); bottom channels (red area) = somatosensory cortex (SMC).

Measurements were performed before and after an 8-week home intervention using a wrist-driven 3D printed transitional hand prosthesis (Fig. 2). Oxygenated haemoglobin as calculated from functional near-infrared spectroscopy (fNIRS) data recordings were indirect indicators of cortical activity during a gross manual dexterity task. Functionality was assessed via performance score on the box and blocks test.

### Participants

Participants were recruited from the community via outreach to clinicians and support groups, existing databases and health record systems. Inclusion criteria for all participants included a paediatric population with congenital ULRD. Wrist and elbow range of motion of the affected limb had to be greater than 20° in order to activate the body-powered prosthesis. Exclusion criteria included upper extremity injury within the past month and any medical conditions that would contraindicate the use of the transitional prosthesis, such as skin abrasions and musculoskeletal injuries.

Fourteen participants (Table 1) with congenital upper-limb deficiencies participated in this study and were fitted with a wrist- or elbow-driven 3D printed prosthesis (Fig. 2A). A sex- and age-matched control group were recruited to participate in this study and were fitted with a matched wrist- or elbow-driven 3D printed prosthetic simulator on their non-dominant hand (Fig. 2A). The prosthetic fitting was performed by a certified prosthetic and orthotic professional. Data collections and prosthetic fitting occurred in the Biomechanics Research Building at the University of Nebraska at Omaha from January 2020 to December 2022. This study was powered to detect a between-group difference of performance in a gross manual dexterity test and brain lateralization^15^ using the free to use software G*Power. Covariant parameters and ULRD group changes were identified from a previous case study.^21^ Calculations indicted a total target sample size of 40. Considering the effect size from preliminary data and to account for a 10% drop-out rate, a total sample of 40 subjects was determined to provide 80% power to detect a true standardized effect size. These calculations were made before any analysis of outcome data.

**Table 1:** Baseline participant characteristics

Characteristics	Participants	
	ULRD (*n* = 14)	Control (*n* = 14)
Age, mean (SD), year	11.1 (3.8)	11.1 (3.8)
Sex		
Male	8 (57.1)	8 (57.1)
Female	6 (42.9)	6 (42.9)
Race		
American Indian	0	0
Asian	5 (35.7)	2 (14.3)
Black/African American	0	1 (7.1)
White	9 (64.3)	11 (78.6)
Hispanic/latinx ethnicity	0	0
Limb (prosthesis/simulator)	Affected	Non-dominant
Left	6 (42.3)	12 (85.7)
Right	8 (57.1)	2 (14.3)
Limb (intact/non-simulator)	Non-affected	Dominant
Left	8 (57.1)	2 (14.3)
Right	6 (42.3)	12 (85.7)
Level of limb reduction		
Partial carpal	11 (78.6)	0
Trans-radial	3 (21.4)	0

Data are presented as the number (percentage) of participants. When describing the limbs of each group, the limbs of ULRD group are characterized as affected limb and non-affected limb, while the limbs of the Control group are characterized as non-dominant and dominant limb. The affected limb of ULRD group utilized the prosthesis while the non-dominant limb of the Control group utilized the prosthetic simulator.

### Prosthesis home intervention

After screening and baseline testing, eligible participants participated in 8 weeks of prosthetic use training in the comfort of their own homes. The home intervention protocol followed previously reported procedures assessing paediatric functional motor skills utilizing a prosthesis,^22^ which have shown significant improvements in gross manual dexterity, bimanual coordination and the functional activities performed during the intervention. Participants participated in two training sessions per week lasting for approximately 1 h for 8 weeks over video conference with the research personnel who were blinded to the aim of the study.

During the prosthesis home intervention, participants completed activities to assess functional completion of activities of daily living with the prosthesis under the direction of the research personnel. All activities were executed by telling the participant, ‘Ready, Set, Go’ and ending with ‘Stop’ with the exception of the drawing activity. The activities included Block Building, Utensils, Paper Activities, Ball Play, Tray Carry and Bike Circuit.^22^

The Utensils, Paper Activity, Ball Play and Tray Carry were considered training tasks, as they occurred during each training session. Utensils consisted of using the participant’s unaffected extremity and the prosthetic appendage to make four cuts with a fork and knife while utilizing a universal cuff to grasp the utensil in the prosthesis. Paper Activities consisted of several parts, stabilizing a piece of paper while the participant draws on it, folding the piece of paper, stabilizing a tape dispenser with the prosthesis to tear three pieces of tape, and holding the paper with the prosthesis while the able hand uses scissors to make three cuts. During Ball Play, the participant must use the prosthesis to pick up a ball from the ground and either underhand or overhand toss it to a partner three times in a row. During Tray Carry, participants walked in a Figure 8 formation around two toys placed approximately 5 ft apart while balancing a tray loaded with two cups between their prostheses and unaffected upper extremity.

The Block Building and Bike Circuit activities were considered non-training tasks and only completed during the first and last weeks of the prosthesis home intervention to assess the pre- and post-intervention abilities. The Block Building activities included two trials of a series of six different Block Building activities for each hand separated by 30 s of rest (a total of 18 block building activities per hand). Specifically, the participant was instructed to perform three trials of the following building block activities: 4-block train, 3-cube bridge, 4-block wall, 3-block tower, 6-block steps and 6-block pyramid. The Bike Circuit required participants to either ride a bike or walk a bike in a Figure 8 around two stationary objects approximately 5 ft apart to demonstrate independent bilateral forearm activation in order to turn the bike with the able appendage and the prosthesis.

### Printed body-driven prosthesis characteristics

The Cyborg Beast 2 (Fig. 2A) was utilized in this study and manufactured in the Additive Manufacturing Laboratory located in the Biomechanics Research Building at the University of Nebraska at Omaha. Materials, modelling software, 3D printers and building components have been previously reported.^5-8,14,15^

### Gross manual dexterity test

The Box and Block Test is a measure of unilateral gross dexterity (Fig. 2B).^23,24^ During the task, the participants were instructed to move one block at a time, transporting it over the partition and dropping it in the opposite compartment. The participants were instructed to move as many blocks as possible within a 60 s period. This was followed by 60 s of rest. Three trials were performed for each limb condition (affected using the prosthetic and non-affected for ULRD group; non-dominant using the simulator and dominant for control group). The number of blocks moved was recorded for each trial.

### Hemodynamic response measurements

Hemodynamic response data were collected using a continuous wave fNIRS system (NIRSport 2, NIRx Medical Technologies, LLC, Berlin, Germany) and Aurora fNIRS–NIRSport 2 Acquisition Software (version 2020.7.2.0). Data were sampled at 8 Hz operating at 760 and 850 nm wavelengths. fNIRS data were collected during the Box and Blocks Test for each limb condition performing the task. The Aurora fNIRS software allowed for an automated signal optimization algorithm to be used during signal calibration which ensured and served as an early-stage signal quality check before data were recorded. Eight sources and eight detectors were placed in a cap fitted to each participant’s head circumference (Fig. 2C). The cap was positioned on the head following the 10–20 international system and the probes were placed according to a standardized montage available through the NIRx support portal (NIRSite Montage Motor 8 × 8).^25^ The fNIRS channels covered the area around the C3 and C4 landmarks which have been shown to detect motor activity that drives hand and arm movement.^26^

### Statistical analysis

The AnalyzIR Toolbox^27^ was used to analyze the fNIRS data. The raw fNIRS signals were first converted into changes in optical density data by taking the logarithm of the signal. Optical density data were then filtered with a fourth order bandpass filter (high pass = 0.01 Hz; low pass = 0.2 Hz) in order to remove for any physiological noise.^28^ The oxygenated-hemoglobin (HbO) concentrations were then obtained using the modified Beer–Lambert law. The hemodynamic response function was then estimated by a general linear model (GLM) approach that uses autoregressive iterative reweighted least squares, which corrected for motion artifacts.^29^ The response was modelled using the Canonical temporal basis function with a peak time of 4 s. Beta values (i.e. changes in HbO) were calculated during the GLM analysis and represent the weighted responses of the individual channels during the task compared to baseline levels. For whole-brain analysis, Student’s *t*-statistic estimates and Benjamini–Hochberg false-discovery rate corrected *P*-values (*q*-values) were also calculated, which addresses the problem of evaluated type 1 error due to multiple comparisons. A significance level of *q* < 0.05 was used in heatmaps of T-scores plotted in 10–20 format.

For analysis of the Box and Blocks Test, a two-way repeated measures ANOVA [2 × 4; group (ULRD versus Control) × hand (affected limb using prosthesis versus non-dominant limb using simulator versus non-affected limb versus dominant limb)] was used to test group versus hand interactions. A Student’s *t*-statistic was utilized to determine between group differences. A *P*-value of less than 0.05 was considered statistically significant for all comparisons.

## Results

### Recruitment and retention

We consented and screened a total of 40 participants who were eligible for this non-randomized controlled trial. However, a total of 28 participants were included in the final data analysis presented in this manuscript, and the demographics are displayed in Table 1. A total of 12 participants were excluded from the final analysis due to the following reasons: (1) participants (*n* = 1) did not complete the full 8 weeks of training; (2) participants (*n* = 7) withdrew from a data collection session, baseline or follow-up, or the data set was considered incomplete; (3) the inclusion criteria of an age- and sex-matched control was not met (*n* = 4).

### Box and Blocks Test

There was a significant interaction between-groups for gross manual dexterity performance, *F*(1,7) = 134.25, *P* < 0.0001. Post-hoc analyses revealed that during the baseline assessment the affected limb using the prosthesis of the ULRD group performed similarly (*P* = 0.9668) to the non-dominant limb using the simulator of the Control group. Likewise, during baseline assessment, the non-affected limb of the ULRD group performed similarly to the dominant limb of the Control group (*P* = 0.6098).

After the completion of the home intervention, these between-group comparisons did not change. During the post-assessment, the affected limb using the prosthesis of the ULRD group performed similarly (*P* = 0.9298) to the non-dominant limb using the simulator of the Control group. Likewise, the non-affected limb of the ULRD group performed similarly to the dominant limb of the Control group (*P* = 0.1249) during the post-assessment.

In agreement with our hypothesis, there was a significant improvement due to the home intervention. After intervention (Table 2), there was a significant improvement in gross manual dexterity within the ULRD group of the affected limb using the prosthesis (*P* = 0.0264) and the non-affected limb (*P* = 0.0003). This significant improvement in gross manual dexterity was also seen within the TD group using the simulator (*P* = 0.0329) and the dominant limb (*P* = 0.0111).

**Table 2:** Average (mean ± SD) number of blocks per minute moved during the box and blocks test

	ULRD group				Control group			
	Affected limb using prosthesis		Non-affected limb		Non-dominant limb using simulator		Dominant limb	
	Baseline	Post	Baseline	Post	Baseline	Post	Baseline	Post
Mean	7.24	12.33^a^	41.43	49.79^a^	7.14	12.13^a^	40.26	46.21**^a^**
SD	4.08	5.57	16.46	15.34	4.74	4.38	11.61	11.90

^a^Significant (*P* < 0.05) increase as compared to within-group/hand baseline measures.

### Areas of cortical activation

**Figure 3:**
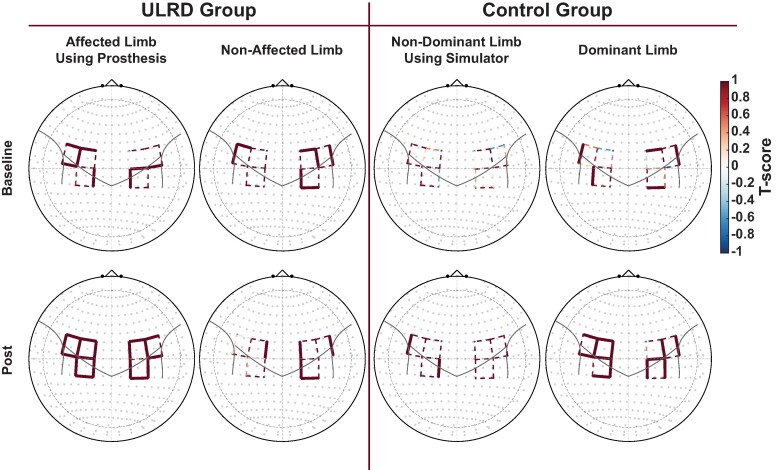
**Cortical activity during the Box and Block Test.** ΔHbO activation maps reflecting T-scores heatmaps for each group and limb. Solid lines are statistically significant contrasts surviving correction for multiple comparisons (*q* < 0.05). Dashed lines are not statistically significant. ULRD, upper-limb reduction deficiency.

Cortical activation maps for each group are presented in Fig. 3. Channels that produced a statistical significance of cortical activation surviving multiple comparisons correction (*q* < 0.05) and a positive T-score (T-score > 0.0) were considered to contain significant cortical activation and are depicted in solid lines.

Before the home intervention, significant cortical activation was produced in all groups and conditions except in the non-dominant limb using the simulator of the Control group. When active, various channels over the left premotor cortex were always significantly active. However, the channels over the right premotor cortex were only significantly active when intact limbs performed the Box and Blocks test. Additionally, various channels over the primary motor cortex were significantly active in both hemispheres within each group, except the aforementioned Control group. Lastly, similar significant activities were produced in the sensory cortex, except in the non-affected limb of the ULRD group.

After the home intervention, a greater number of channels were significantly active. Widespread changes were especially seen in the affected limb using the prosthesis of the ULRD group. Most channels became significantly active with the exception of one channel (S5, D7) located in the right primary motor cortex.

### Effects of intervention on oxygenated hemodynamic responses

**Figure 4:**
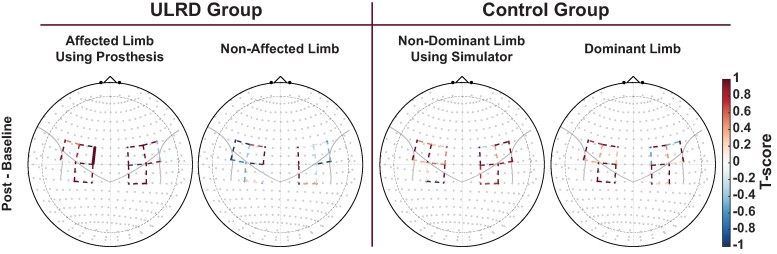
**Changes in cortical activity due to intervention.** ΔHbO activation maps reflecting post-baseline contrast T-scores for each group and limb performing the Box and Block Test. Solid lines are statistically significant contrasts surviving correction for multiple comparisons (*q* < 0.05). Dashed lines are not statistically significant. ULRD, upper-limb reduction deficiency.

Comparisons of ΔHbO responses for each group before and after intervention are presented in Fig. 4. The home intervention produced a significant increase in the ΔHbO recorded from one channel (S1, D1) located in the left premotor cortex. This significant increase was only seen in the affected limb using the prosthesis of the ULRD group (*q* = 0.0042). There were no significant changes in ΔHbO in the non-affected limb of the ULRD group nor in either limb of the Control group (*q* > 0.05).

### Adverse events

There were no serious adverse events related to study procedures to report.

## Discussion

The novel finding of this longitudinal clinical trial partially agrees with our hypothesis that the home intervention would cause robust changes to the sensorimotor cortex (Fig. 3). However, the changes seen after 8 weeks of prosthesis home intervention were only restricted to the left premotor cortex of the ULRD group when using the prosthesis with the affected limb (Fig. 4). Secondary findings agree with the hypothesis indicating a non-significant difference in gross manual dexterity between prosthetic and prosthetic simulator groups and between the non-affected and dominant hand groups before the onset of the home intervention (Table 2).

### Gross manual dexterity in terms of handedness

The results of the Box and Blocks Test were in agreement with our previous studies that also showed no significant difference in gross manual dexterity between the ULRD group using the prosthesis and typically developing Control group using the simulator.^15^ However, the non-significant difference in gross manual dexterity between the ULRD’s non-affected limb and Control’s dominant limb disagrees with previously reported performances that showed the ULRD group performed significantly lower than the Control group during the Box and Blocks Test.^15^

Sainburg *et al.* have indicated that the dominant-arm system controls movement extent largely through planning mechanisms that specify ‘open-loop’ control, while the non-dominant arm system does so largely through feedback mechanisms or ‘closed-loop’ control.^30,31^ In the context of this study, the affected limb of the ULRD group was assumed to be non-dominant limb; however, the handedness of the affected limb was not utilized as a covariant. After intervention, both limbs in each group performed significantly better during the Box and Blocks Test (Table 2). Since the intervention improved performance of both limbs within the ULRD group, these results suggest that the intervention provided a profound benefit on both the planning (open-loop) as well as the feedback (closed-loop) systems of the ULRD group regardless of the handedness of the affected limb.

### Interpretation and generalization of induced cortical plasticity

The baseline activation maps produced during the Box and Blocks Test in the current study (Fig. 3) agree with our previous report where it was shown that ULRD children, unlike a typically developing Control group, showed significant activation in both hemispheres.^15^ This bi-lateral response is also consistent with recent findings in adults with acquired amputation^32,33^ and unilateral stroke.^17,18,20,34-36^ However, it has been suggested that children may lack lateralization in the early stages.^37^ More importantly, this bi-lateral response has also been shown in healthy adults during unilateral finger movement^38,39^ and unilateral arm movements,^40-42^ which agree with the activation maps of the Control group using the dominant limb. After intervention, a greater number of channels became active in the ULRD group. This may suggest that the intervention produced large-scale inter-network communication across hand-selective areas, which has been reported in amputees utilizing a prosthesis daily.^43^ Thus, it is reasonable to suggest if the child uses the prosthesis extensively, it would facilitate the representation of the arterial limb on areas of the motor cortex normally devoted to the missing limb thus facilitating prosthesis acceptance and embodiment.^10,12,15^

Due to these results showing bi-lateral activation during unilateral limb movements, it has been suggested that there is a functional role for each hemisphere.^44^ For motor function, it has been suggested that the left hemisphere controls predicting and accounting for limb dynamics while the right hemisphere controls stabilizing limb position through impedance control mechanisms.^16,45^ For example, it has been shown in right handed individuals that left, dominant hemisphere damage produced deficits in intersegmental coordination and in direction learning during adaptation to visuomotor rotation, whereas right, non-dominant hemisphere damage produced deficits in final position accuracy and position adaptation.^17,18,20^

According to the neuronal group selection theory, the ULRD population may lack representation of the missing part of the limb in the cerebral cortex.^10,12,15,46^ Thus, one could speculate that the ULRD group, with a congenital as opposed to acquired limb reduction, would show most control deficits related to the affected hemisphere. Since intersegmental coordination and final position accuracy and adaptation were not specifically measured in this study, we will focus on the increase in HbO response of the left premotor cortex (Fig. 4) due to the intervention. This result suggests that the intervention may have improved the functional role of the left hemisphere which translated to the improvement of learning direction during adaptation to visuomotor control.^15,34,47^ In the current study, an improvement in visuomotor control was reported as an improvement in the gross manual dexterity (Box and Blocks) test after intervention (Table 2), where the use of the prosthesis and home intervention was assumed to provide closed-loop training. In terms of hemisphere specificity, the results suggest that the intervention had a profound effect on the contralateral (left, dominant) hemisphere of the paediatrics with an affected right limb but on the ipsilateral (left, non-dominant) hemisphere of the paediatrics with an affected left limb. This would mean that the intervention provided different benefits based on the handedness of the affected limb and the corresponding dominant or non-dominant hemisphere. Since the prosthesis could be considered to initiate closed-loop training for the affected limb, we could suspect that the home intervention may be more beneficial for ULRD paediatrics with a reduction of the left limb.

### Limitations and future considerations

The main limitations of the present study are related to the smaller number of paediatrics with ULRD participating in this study (*n* = 14), larger age range (6–18 years of age), the inclusion of different reduction levels, including partial carpal (*n* = 11) and *trans*-radial (*n* = 3) reductions, and not separating the ULRD group based on the affected limb, left or right side of the body. To partially control for these limitations, the Control group was age- and sex-matched and utilized a prosthetic simulator equivalent to the prosthesis utilized by the ULRD group.

Future studies should include additional coordination tasks in order to connect a specific function to the respective hemisphere as well as resting-state fNIRS measures to measure inter-network communication. In addition, future studies should utilize the side of the body of the affected limb as a covariant as the affected limbs were not separated in this study due to the smaller population size.

## Conclusion

We have demonstrated that an 8-week prosthesis home intervention provided benefit to paediatrics with ULRD. The observed behavioural improvements and cortical plasticity may suggest a functional role of the left hemisphere to improve performance as well as the potential embodiment of a prosthetic device in the cerebral cortex after extensive prosthesis training.

## Data Availability

The raw data supporting the conclusions of this article will be made available by the authors, without undue reservation.

## References

[1] CDC . Facts about Upper and Lower Limb Reduction Defects. Accessed February 2021, https://www.cdc.gov/ncbddd/birthdefects/ul-limbreductiondefects.html

[2] Giele H, Giele C, Bower C, Allison M. The incidence and epidemiology of congenital upper limb anomalies: A total population study. J Hand Surg Am. 2001;26(4):628–634. 11466636 10.1053/jhsu.2001.26121

[3] Canfield MA, Honein MA, Yuskiv N, et al National estimates and race/ethnic-specific variation of selected birth defects in the United States, 1999–2001. Birth Defects Res A Clin Mol Teratol. 2006;76(11):747–756. 17051527 10.1002/bdra.20294

[4] Ten Kate J, Smit G, Breedveld P. 3D-printed upper limb prostheses: A review. Disabil Rehabil Assist Technol. 2017;12(3):300–314. 28152642 10.1080/17483107.2016.1253117

[5] Zuniga J, Katsavelis D, Peck J, et al Cyborg beast: A low-cost 3d-printed prosthetic hand for children with upper-limb differences. BMC Res Notes. 2015;8:10. 25601104 10.1186/s13104-015-0971-9 PMC4304188

[6] Zuniga JM, Carson AM, Peck JM, Kalina T, Srivastava RM, Peck K. The development of a low-cost three-dimensional printed shoulder, arm, and hand prostheses for children. Prosthet Orthot Int. 2017;41(2):205–209. 27117013 10.1177/0309364616640947

[7] Zuniga JM, Peck J, Srivastava R, Katsavelis D, Carson A. An open source 3D-printed transitional hand prosthesis for children. J Prosthet Orthot. 2016;28(3):103–108.

[8] Zuniga JM, Young KJ, Peck JL, et al Remote fitting procedures for upper limb 3d printed prostheses. Expert Rev Med Devices. 2019;16(3):257–266. 30661413 10.1080/17434440.2019.1572506

[9] Hadders-Algra M, Reinders-Messelink HA, Huizing K, van den Berg R, van der Sluis CK, Maathuis CG. Use and functioning of the affected limb in children with unilateral congenital below-elbow deficiency during infancy and preschool age: A longitudinal observational multiple case study. Early Hum Dev. 2013;89(1):49–54. 22863184 10.1016/j.earlhumdev.2012.07.011

[10] Huizing K, Reinders-Messelink H, Maathuis C, Hadders-Algra M, van der Sluis CK. Age at first prosthetic fitting and later functional outcome in children and young adults with unilateral congenital below-elbow deficiency: A cross-sectional study. Prosthet Orthot Int. 2010;34(2):166–174. 20298129 10.3109/03093640903584993

[11] Biddiss EA, Chau TT. Upper limb prosthesis use and abandonment: A survey of the last 25 years. Prosthet Orthot Int. 2007;31(3):236–257. 17979010 10.1080/03093640600994581

[12] Meurs M, Maathuis CG, Lucas C, Hadders-Algra M, van der Sluis CK. Prescription of the first prosthesis and later use in children with congenital unilateral upper limb deficiency: A systematic review. Prosthet Orthot Int. 2006;30(2):165–173. 16990227 10.1080/03093640600731710

[13] Zuniga JM, Dimitrios K, Peck JL, et al Coactivation index of children with congenital upper limb reduction deficiencies before and after using a wrist-driven 3D printed partial hand prosthesis. J Neuroeng Rehabil. 2018;15(1):48. 29884185 10.1186/s12984-018-0392-9 PMC5994003

[14] Zuniga JM, Peck JL, Srivastava R, et al Functional changes through the usage of 3D-printed transitional prostheses in children. Disabil Rehabil Assist Technol. 2019;14(1):68–74. 29116866 10.1080/17483107.2017.1398279 PMC5940585

[15] Zuniga JM, Pierce JE, Copeland C, et al Brain lateralization in children with upper-limb reduction deficiency. J Neuroeng Rehabil. 2021;18(1):24. 33536034 10.1186/s12984-020-00803-1 PMC7860186

[16] Sainburg RL . Handedness: Differential specializations for control of trajectory and position. Exerc Sport Sci Rev. 2005;33(4):206–213. 16239839 10.1097/00003677-200510000-00010 PMC10709818

[17] Schaefer SY, Haaland KY, Sainburg RL. Ipsilesional motor deficits following stroke reflect hemispheric specializations for movement control. Brain. 2007;130(Pt 8):2146–2158. 17626039 10.1093/brain/awm145 PMC3769213

[18] Schaefer SY, Haaland KY, Sainburg RL. Hemispheric specialization and functional impact of ipsilesional deficits in movement coordination and accuracy. Neuropsychologia. 2009;47(13):2953–2966. 19573544 10.1016/j.neuropsychologia.2009.06.025 PMC2752301

[19] Schaefer SY, Haaland KY, Sainburg RL. Dissociation of initial trajectory and final position errors during visuomotor adaptation following unilateral stroke. Brain Res. 2009;1298:78–91. 19728993 10.1016/j.brainres.2009.08.063 PMC3151492

[20] Mani S, Mutha PK, Przybyla A, Haaland KY, Good DC, Sainburg RL. Contralesional motor deficits after unilateral stroke reflect hemisphere-specific control mechanisms. Brain. 2013;136(Pt 4):1288–1303. 23358602 10.1093/brain/aws283 PMC3613707

[21] Borrell JA, Copeland C, Lukaszek JL, Fraser K, Zuniga JM. Use-dependent prosthesis training strengthens contralateral hemodynamic brain responses in a young adult with upper limb reduction deficiency: A case report. Front Neurosci. 2021;15:693138. 34177460 10.3389/fnins.2021.693138 PMC8226211

[22] Lukaszek JL, Borrell JA, Cortes C, Zuniga JM. Home intervention for children and adolescents with unilateral trans-radial and partial carpal reduction deficiencies. Sci Rep. 2022;12(1):7447. 35523915 10.1038/s41598-022-11470-8 PMC9076824

[23] Mathiowetz V, Volland G, Kashman N, Weber K. Adult norms for the box and block test of manual dexterity. Am J Occup Ther. 1985;39(6):386–391. 3160243 10.5014/ajot.39.6.386

[24] Mathiowetz V, Wiemer DM, Federman SM. Grip and pinch strength: Norms for 6- to 19-year-olds. Am J Occup Ther. 1986;40(10):705–711. 3777107 10.5014/ajot.40.10.705

[25] Klem GH, Lüders HO, Jasper HH, Elger CE. The ten-twenty electrode system of the international federation. The international federation of clinical neurophysiology. Electroencephalogr Clin Neurophysiol Suppl. 1999;52:3–6. 10590970

[26] Nishiyori R, Bisconti S, Ulrich B. Motor cortex activity during functional motor skills: An fNIRS study. Brain Topogr. 2016;29(1):42–55. 26243304 10.1007/s10548-015-0443-5

[27] Santosa H, Zhai X, Fishburn F, Huppert T. The NIRS brain AnalyzIR toolbox. Algorithms. 2018;11(5):73. 38957522 10.3390/a11050073 PMC11218834

[28] Pinti P, Scholkmann F, Hamilton A, Burgess P, Tachtsidis I. Current status and issues regarding pre-processing of fNIRS neuroimaging data: An investigation of diverse signal filtering methods within a general linear model framework. Front Hum Neurosci. 2019;12:505. 30687038 10.3389/fnhum.2018.00505 PMC6336925

[29] Barker JW, Aarabi A, Huppert TJ. Autoregressive model based algorithm for correcting motion and serially correlated errors in fNIRS. Biomed Opt Express. 2013;4:1366–1379. 24009999 10.1364/BOE.4.001366 PMC3756568

[30] Sainburg RL, Schaefer SY. Interlimb differences in control of movement extent. J Neurophysiol. 2004;92(3):1374–1383. 15115793 10.1152/jn.00181.2004 PMC3769166

[31] Sainburg RL . Evidence for a dynamic-dominance hypothesis of handedness. Exp Brain Res. Jan 2002;142(2):241–258. 11807578 10.1007/s00221-001-0913-8 PMC10710695

[32] Philip BA, Frey SH. Compensatory changes accompanying chronic forced use of the nondominant hand by unilateral amputees. J Neurosci. 2014;34(10):3622–3631. 24599461 10.1523/JNEUROSCI.3770-13.2014 PMC3942579

[33] Hamzei F, Liepert J, Dettmers C, et al Structural and functional cortical abnormalies after upper limb amputation during childhood. Neuroreport. 2001;12(5):957–962. 11303768 10.1097/00001756-200104170-00019

[34] Mutha PK, Sainburg RL, Haaland KY. Left parietal regions are critical for adaptive visuomotor control. J Neurosci. 2011;31(19):6972–6981. 21562259 10.1523/JNEUROSCI.6432-10.2011 PMC3107546

[35] Mutha PK, Sainburg RL, Haaland KY. Critical neural substrates for correcting unexpected trajectory errors and learning from them. Brain. 2011;134(12):3647–3661. 22075071 10.1093/brain/awr275 PMC3235559

[36] Schaefer SY, Mutha PK, Haaland KY, Sainburg RL. Hemispheric specialization for movement control produces dissociable differences in online corrections after stroke. Cereb Cortex. 2012;22(6):1407–1419. 21878488 10.1093/cercor/bhr237 PMC3357180

[37] Marcori AJ, Teixeira LA, Dascal JB, Okazaki VHA. Are the predictions of the dynamic dominance model of laterality applicable to children? Dev Neuropsychol. 2020;45(7–8):496–505. 33203247 10.1080/87565641.2020.1849220

[38] Li A, Yetkin FZ, Cox R, Haughton VM. Ipsilateral hemisphere activation during motor and sensory tasks. AJNR Am J Neuroradiol. 1996;17(4):651–655. 8730183 PMC8337268

[39] Cramer SC, Finklestein SP, Schaechter JD, Bush G, Rosen BR. Activation of distinct motor cortex regions during ipsilateral and contralateral finger movements. J Neurophysiol. 1999;81(1):383–387. 9914297 10.1152/jn.1999.81.1.383

[40] Nirkko AC, Ozdoba C, Redmond SM, et al Different ipsilateral representations for distal and proximal movements in the sensorimotor cortex: Activation and deactivation patterns. Neuroimage. 2001;13(5):825–835. 11304079 10.1006/nimg.2000.0739

[41] Winstein CJ, Grafton ST, Pohl PS. Motor task difficulty and brain activity: Investigation of goal-directed reciprocal aiming using positron emission tomography. J Neurophysiol. 1997;77(3):1581–1594. 9084621 10.1152/jn.1997.77.3.1581

[42] Sainburg RL, Kalakanis D. Differences in control of limb dynamics during dominant and nondominant arm reaching. J Neurophysiol. 2000;83(5):2661–2675. 10805666 10.1152/jn.2000.83.5.2661 PMC10709817

[43] van den Heiligenberg FMZ, Orlov T, Macdonald SN, et al Artificial limb representation in amputees. Brain. 2018;141(5):1422–1433. 29534154 10.1093/brain/awy054 PMC5917779

[44] Gazzaniga MS . Cerebral specialization and interhemispheric communication: Does the corpus callosum enable the human condition? Brain. 2000;123(Pt 7):1293–1326. 10869045 10.1093/brain/123.7.1293

[45] Yadav V, Sainburg RL. Motor lateralization is characterized by a serial hybrid control scheme. Neuroscience. 2011;196:153–167. 21889579 10.1016/j.neuroscience.2011.08.039 PMC3199140

[46] Sporns O, Edelman GM. Solving Bernstein's problem: A proposal for the development of coordinated movement by selection. Child Dev. 1993;64(4):960–981. 8404271

[47] Wang J, Sainburg RL. Mechanisms underlying interlimb transfer of visuomotor rotations. Exp Brain Res. 2003;149(4):520–526. 12677333 10.1007/s00221-003-1392-x PMC3697093

